# A Predictive Distribution Map for the Giant Tropical Ant, *Paraponera clavata*

**DOI:** 10.1673/031.007.0801

**Published:** 2007-02-01

**Authors:** Christina M. Murphy, Michael D. Breed

**Affiliations:** Department of Ecology and Evolutionary Biology, The University of Colorado, Boulder

## Abstract

*Paraponera clavata* (Fabricius 1775) (Formicidae: Paraponerinae) is a widely distributed Neotropical ant whose large size has attracted the attention of numerous collectors. Working from museum specimens, a georeferenced database of collection localities was developed. This database then served as the source for computer generated predictive distribution maps. Annual rainfall was the most important variable chosen by the computer model to predict the distribution of *P. clavata*, both on the scale of the neotropics and at a finer scale at the northern end its distribution in Costa Rica and Nicaragua. When the model was forced to use vegetation as the first predictive variable, the Neotropical model used temperature and rainfall variance as additional variables, while the Mesoamerican model used both climatic and soils variables. Overall, the modeling suggests that *P. clavata* is more sensitive to abiotic factors (rainfall, temperature, soils) than to biotic factors (vegetation type) in its distribution, although this conclusion comes with the caveat that the vegetation types used in the model are quite generalized. Predictive distribution mapping holds great promise for generating more precise representations of insect distributions, thereby allowing better tests of the extent of distribution overlaps and other community relationships.

## Introduction

The giant tropical ant, *Paraponera clavata* (Fabricius 1775) (Formicidae: Paraponerinae), is one of the most visible Neotropical insects. It is widely distributed, having been collected from Nicaragua in the north to Bolivia and Brazil at its southern limit. Because of the abundance of this species in some habitats, its visual prominence, and a well-deserved reputation for its painful sting, *P. clavata* has been more generally collected than most insect species. Taxonomically, *P. clavata* is the monotypic member of the ant subfamily Paraponerinae (Bolton 2003). Its systematic isolation from other ants makes this species a particularly interesting object of study. While investigators have long recognized its wide distribution in Neotropical wet forests (Janzen and Carroll 1983), no formal study of its distribution has been undertaken.

Studies of *P. clavata* nest distribution have focused on association of nests with plant species at two Mesoamerican sites, La Selva Biological Station in Costa Rica (Bennett and Breed 1985; Breed et al. 1991; Dyer 2002) and Barro Colorado Island in Panama (Belk et al. 1989, Thurber et al. 1993; Perez et al. 1999). *P. clavata* typically nests in soil at the base of rainforest trees, although nests have also been reported in soil accumulations in tree crotches (Breed and Harrison 1989) and in at least one case in treeless *Campo Cerrado*, dry tropical savanna interspersed with woody shrubs, (Brazil; C. R. F. Brandão, Musuem of Zoology, University of San Paolo, Brasil, personal communication). These studies add to our knowledge of preferred habitats at a local level, but cannot be scaled to regional or continental levels because of differences in tree species composition and vegetative structure across wet tropical forests in the neotropics.

Distribution maps are valuable tools for assessing community relationships and beta diversity. Usually investigators create such maps by drawing outlines that encompass known collection points; oftentimes the distribution outline relies on relatively few data points and is primarily based on the intuition of the investigator. A map may encompass large areas of unsuitable habitat because its creator focuses on distribution boundaries, rather than occurrence within those boundaries. Alternatively, computational geographic modeling systems give tools for using collection data to make distribution predictions based on quantitative assessments of niche characteristics (Stockwell and Noble 1992, Stockwell and Peters 1999, Stockwell and Peterson 2002, Stockwell and Peterson 2003, Stockwell 2006).

A distribution map for *P. clavata* is important for a number of reasons. It is one of the better-known Neotropical ants; its large size and broad distribution give a predictive distribution map for this species considerable interest. The relatively high abundance of nests and large biomass of the ants within those nests, at least in some habitats, makes *P. clavata* a major consumer in some tropical forests, on a par with some bird and mammal species (Tillberg and Breed 2004). We had two objectives; first, an ecological niche modeling system, WhyWhere (Stockwell 2004), was used to create probabilistic distribution maps for *P. clavata*, and second, primary niche requirements for *P. clavata* were characterized using the GIS data layers that best described its distribution.

## Methods

### Data collection

Locality points for *Paraponera clavata* that were used to build the distributional models came from location information from the label data of museum specimens. Several hundred museum specimens (American Museum of Natural History; US National Museum; Los Angeles County Museum of Natural History; University of Colorado; Museum de Zoologia, Sao Paolo; Instituto Nacional de Biodiversidad, Costa Rica; DiGIR Service, Ant Collection Catalog, California Academy of Sciences) yielded 186 georeferenced latitude-longitude coordinates. All locality points that did not have latitude-longitude information with the specimen were georeferenced using online mapping resources. A randomly chosen subset of 37 Costa Rican points from INBIO and other sources was used in the models. Use of all the Costa Rican points in the Neotropical models would have overwhelmed the information from other parts of the range; use of this subset for Costa Rica ensured an appropriate geographic balance in constructing the model. For all locations, the number of points is far fewer than the number of specimens because many specimens were collected from the same localities, reflecting patterns of access by collectors.

### Analysis

The analysis was performed using the WhyWhere 2.0 ecological niche modeling application (Stockwell 2004), a project supported at the San Diego Supercomputer Center. WhyWhere allows selection among several databases for use in modeling ecological distributions. The “Annual” database, which has 33 data layers was chosen. The developers of WhyWhere used climatic, vegetational and soils maps from publicly available computerized databases when creating their software. Seven layers are climatic, derived from Legates & Willmott (Willmott et al. 1998) climatic databases. Four other layers are principal components representing climatic gradients, three are from the Olson World Ecosystems database (Allison et al. 2000), seven from the Staub and Rosenzweig Zobler Soil database (Staub and Rosenzweig 1986a, b), five from Wilson & Henderson-Sellers vegetation and soils database (Wilson and Henderson-Sellers 2003), and five soil layers from Webb et al. (2000). Most of these layers have a resolution of 1.0 or 0.5 degrees. Most notably missing is an elevation database, however, the upper and lower elevation bounds of the distribution based on label data are considered below.

WhyWhere uses a subset of data points to generate the model and the remaining points to test the accuracy of the model. Here, 80% of the data points were used to build the model and 20% of the data points were used to test the model. To develop a model, the WhyWhere mapping routine chooses the “training” set of data points and then looks for the layer which best explains their distribution; a second loop is then performed, which adds a second layer to the model. Models were run at a resolution of 0.5 degrees (over the entire range of *P. clavata*) or 0.016667 degrees (for the maps of Costa Rica and Nicaragua). These levels of resolution were adequate to capture the information available in the data layers; long run-times for the models precluded using higher resolutions for the full dataset. However, due to the resolution of the data layers, higher resolution maps would likely have generated no additional information. Because different training datapoints are used in different runs, not all runs on the same dataset yield the same result; multiple runs were used to find the models that yielded the highest accuracy for each dataset. WhyWhere also allows the user to specify a layer to be used first in the model; for each dataset the hypothesis tested was that *P. clavata* distribution follows vegetation type by forcing the first data layer as Olson World Ecosystem Classes (layer 12 in WhyWhere), which contains 59 vegetation categories, mapped at 10 or 30 minute lat-long resolution. When a layer is forced, the routine performs two additional loops, resulting in a model with three layers.

The distribution maps were projected in ArcGIS 9.0, with the original data points overlaid and the symbology revised.

### Costa Rican and Nicaraguan Range

Three Nicaraguan points were added to the 37 Costa Rican points, so a dataset of 40 points was used to predict the distribution of *Paraponera clavata* in its upper range. Most of these points were obtained from the INBIO database; a few were added from collection materials. The Costa Rican distribution was modeled by itself because this country had the greatest concentration data points as Costa Rica has been exhaustively sampled by INBIO parataxonomists, and because the latitude-longitude data were the most accurate for this country. Three points for Nicaragua were added to the Costa Rican data because these represent the northernmost extent of the distribution of *P. clavata* and are geographically proximate to Costa Rica. Finding the predicted distribution of *P. clavata* at the extreme of its range also allowed for testing if the same environmental layers were the best predictors of this species in an area that has been well sampled compared to its entire range.

### Precipitation data and Rainforest distribution

Annual precipitation data were also obtained (WorldClim 2004) and the distribution of vegetation in the neotropics, as used by the model (layer 12; Allison et al. 2000). These data were projected in ArcGIS 9.0 for comparison with the *P. clavata* distribution maps.

## Results

### Elevational records

Collectors found *P. clavata* most commonly in lowland habitats, at elevations from sea level to 750 meters. The highest elevations represented in the sample of specimens used are at 750 meters, near Quincemil in Amazonian Peru, at 1000 meters in the Department of Valle, Colombia, and a single specimen at 1500–1600 meters in Parque La Amistad, in Costa Rica near the Panamanian border on the Atlantic side (Limon Province). The Colombian location, on the western slope of the Andes, is particularly wet, with up to 8 meters of rainfall annually. Only the single record from La Amistad came from above 1500 meters elevation, the lower threshold for upper montane rainforest.

**Figure 1 (A).**
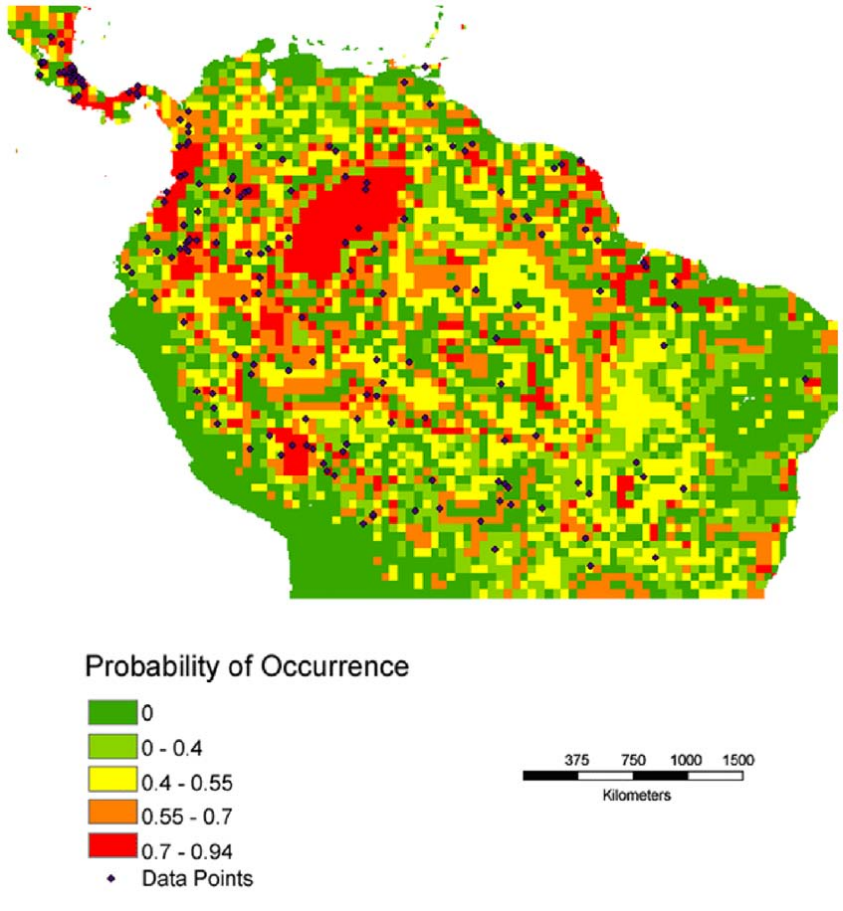
Predictive distribution maps for *P. clavata*. Codes in bold below are from the WhyWhere application. Predicted Neotropical distribution when model chose **Iwcproo,** Legates & Willmott Annual Corrected Precipitation (Willmott et al. 1998). and **wrzsoil,** Webb et al. Soil Particle Size Properties Zobler Soil Types (Staub and Rosenzweig 1986a, b). Accuracy of this model is 0.674. 10.5% of the mapped landmass is in the highest probability class (red on the map, 0.70 and higher probability of occurrence).

### *Paraponera* on the Pacific Slope in Costa Rica

Of the 556 specimens of *P. clavata* in the INBIO database, 29 were collected on the Pacific slope. A lone specimen is attributed to the Osa Peninsula, at 8.40 N, -83.34 W. A group of 13 specimens, all from the same location between Liberia and Cañas, 10.58 N, -85.32 W, a nearby group of 14 specimens from 10.59 N, -85.25 W, and a single specimen from 10.51 N, -85.34 W are also from the Pacific drainage. A cluster of 158 specimens comes from three locations that are equally far west, just south of Lake Nicaragua, but in the Atlantic drainage.

### Predictive mapping

Four predictive distribution maps were generated, based on the entire data set for Central and South America (Figures 1A-B) and on the data for Costa Rica and Nicaragua (Figures 1 C-D). The models for Figures 1A and 1C allowed the model to freely enter variables; in Figures 1B and 1D the model was forced to enter an ecosystem (vegetation type) layer first.

**Figure 1 (B).**
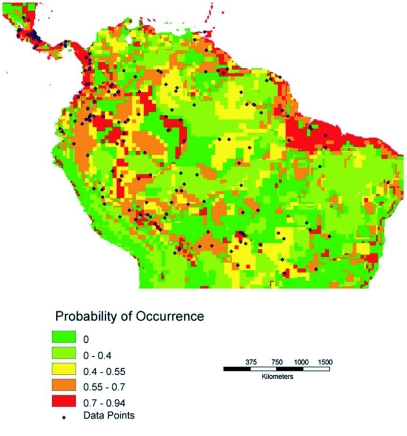
Predictive distribution maps for *P. clavata*. Codes in bold below are from the WhyWhere application. Predicted Neotropical distribution when model is forced to use an ecosystem classification, **owe14d,** Olson World Ecosystem Classes (Allison et al. 2000), as the first layer. Subsequent layers entered into the model were **lwtmpoo,** Legates & Willmott Annual Temperature (Willmott et al. 1998), and **lwerroo** Legates & Willmott Annual Standard Error [rainfall] (Willmott et al. 1998). Accuracy of this model is 0.647. 8.4% of the mapped landmass is in the highest probability class (red on the map, 0.70 and higher probability of occurrence).

The first predictive distribution map for the entire range (Figure 1A) is most strongly influenced by rainfall. In nine of eleven runs the first layer was a rainfall variable and the second layer was a soils layer. In the remaining two cases, both layers were rainfall variables. Accuracies were quite similar among the models; Figure 1A shows the model with the highest accuracy. In all six runs of the model for the neotropics in which the vegetation layer was forced (Figure 1B), rainfall variables entered as the second layer. In three of the cases, rainfall layers were also the third layer, and in the remaining three cases a vegetation layer entered the model.

Interestingly, in ten runs of the model for the Costa Rican data (Figure 1C), seven resulted in the use of two soils variables. In two runs the model used a rainfall variable as the first layer and a soils variable as the second layer; this model had a higher accuracy and is presented in Figure 1C. The remaining run employed two rainfall layers and had lower reliability.

**Figure 1 (C).**
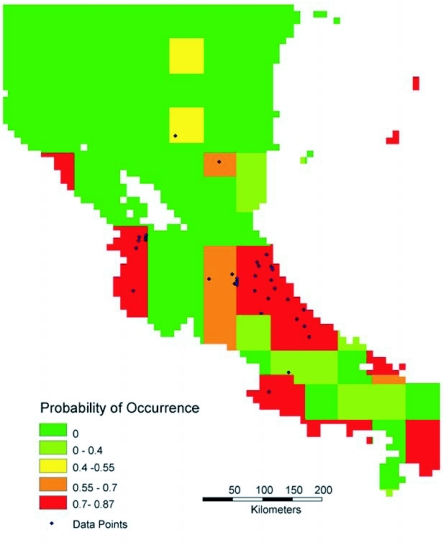
Predictive distribution maps for *P. clavata*. Codes in bold below are from the WhyWhere application. Predicted distribution in Costa Rica and Nicaragua when model chose **lwcproo,** Legates & Willmott Annual Corrected Precipitation (Willmott et al. 1998), and **whsrel,** Wilson & Henderson-Sellers Soil Class Reliability (Wilson and Henderson-Sellers 2003). Accuracy for this model is 0.839.

Forcing a vegetation layer yielded a Costa Rican model that differed substantially from the model that spans the neotropics. The layers used in the Neotropical model, after vegetation, are temperature and rainfall variance (Figure 1B), while soils variables are used in both layers of the Costa Rican model (Figure 1D). In nine of the fifteen runs of the model for Costa Rica in which the vegetation layer was forced (Figure 1D), a rainfall variable was used as the second layer and a soils variable as the third layer. These models were somewhat less accurate than the other six runs, in which two soils variables entered as the second and third layers.

For comparison purposes, a rainfall map for the neotropics is shown in Figure 2A and a distribution map of vegetation types is shown in Figure 2B. Visually there appears to be a strong correspondence between annual rainfall and the predicted distributions in Figures 1A and 1C.

## Discussion

Despite some limitations, a distribution map for *Paraponera clavata* holds considerable interest and ecological niche modeling currently provides the best way to quantitatively create a distribution map (Peterson 2001, Reutter et al. 2003, Wang et al. 2003, Peterson et al. 2004). This predictive mapping confirms *P. clavata* as a typical denizen of wet and moist tropical forest types with relatively even rainfall throughout the year. *P. clavata*'s distribution is correlated with high rainfall, both for the entire range of the species and just for Costa Rica. Other variables generally associated with lowland wet forest—low seasonal variance in rainfall and high temperature—also each enter the models. Soils variables probably gain their importance in three of the models because of the importance of soil in determining vegetation type and nutrient availability to support terrestrial communities. In terms of predictive mapping for the Costa Rican/Nicaraguan subsample, *P. clavata* is sensitive to rainfall, soil texture, and soil moisture. Clearly, vegetation is not independent of these variables, but it is interesting that *P. clavata* maps to these abiotic factors, rather than to the vegetation types available to the modeling software.

**Figure 1 (D).**
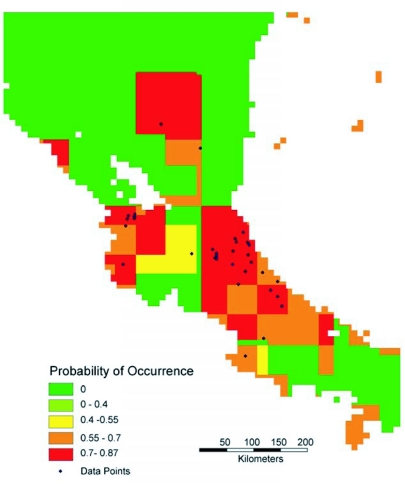
Predictive distribution maps for *P. clavata*. Codes in bold below are from the WhyWhere application. Predicted distribution when model is forced to use an ecosystem classification, **owe14d** Olson World Ecosystem Classes (Allison et al. 2000), as first variable. Subsequent variables entered into the model were **srztext,** Staub and Rosenzweig Zobler Near-Surface Soil Texture (Staub and Rosenzweig 1986a, b), and **wrmodii,** Webb et al. Model II Soil Water (Webb et al. 2000). Accuracy of the model is 0.822.

The most interesting difference between the Neotropical map in which the model chose both variables freely and the map for which vegetation was forced as the first layer is the elimination of the Rio Negro drainage as high probability habitat, when vegetation is considered. This probably corresponds to the low nutrient availability and substantially different forest types found in this drainage. Elevation is also an important factor in limiting *P. clavata* distribution, but elevation is highly correlated with temperature and vegetation type. Because of this, elevation probably only becomes a factor in our models in which a vegetation layer is forced as the first variable. Then, to the extent that vegetation and elevation are correlated, the resulting map reflects an elevational component.

**Figure 2 (A).**
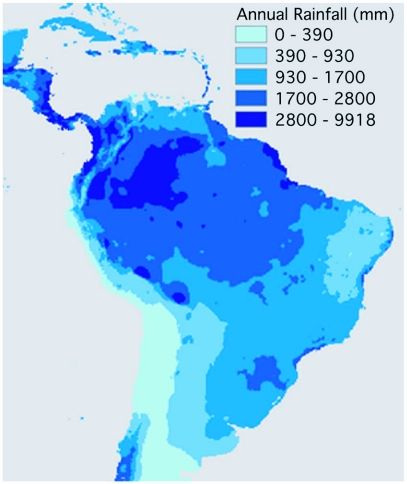
Precipitation map for the neotropics, based on world climate data (Worldclim 2004).

The current limitations of this approach are two-fold. First, the maps are limited by the accuracy and resolution of the underlying datasets. This is apparent in Figures 1C and 1D, which suffer from a poor match between the 1.0 degree or 0.5 degree resolution offered by most of the variables, and the size of the area sampled in Costa Rica and Nicaragua. Second, even though this is a much-collected organism, repetitious collection at given locations (for example, at Barro Colorado Island in Panama or La Selva Biological Station in Costa Rica) inflate the number of museum specimens relative to the number of actual collection points; it was surprising that the final number of georeferenced points was less than 200. More inaccessible areas to biologists are also not well sampled, decreasing the number of localities.

Statements about *P. clavata* like that of Janzen and Carroll (1983): “Oddly, they are absent from the rain forests of the Pacific coast of Costa Rica.” have proven to be incorrect in the light of more extensive collecting. The distribution map displayed in Figure 1 not only predicts that this species occurs on the Pacific side of Costa Rica but shows that this species has actually been collected there as well. The question remains as to why *P. clavata* is so rare on the Pacific side of the continental divide, given the apparent suitability of this habitat.

**Figure 2 (B).**
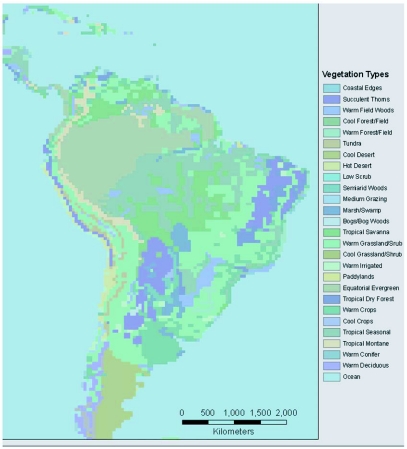
Neotropical vegetation, based on the layer **owe14d** Olson world Ecosystem Classes (Allison et al. 2000); this vegetation data is used as the first layer in the models for Figures 1B and 1D.

These maps are hypotheses, not fixed conclusions. There is clearly good correspondence between the models and the actual distributions in Costa Rica and Nicaragua, which have been more exhaustively sampled. It will be interesting to see if studies in the Amazonian basin and in northern South America are more supportive of the model which predicts a high probability of occurrence in the Rio Negro drainage (Figure 1A) or a low probability in that area (Figure 1B). The models differ in other interesting details, which further collection should also resolve.
